# Supplementary material for: Relationship between chromatin configuration and maturation ability of rat oocytes in vitro and in vivo
Source: PLoS One. 2025 Feb 13;20(2):e0312241. doi: 10.1371/journal.pone.0312241 (PMC11825056; doi:10.1371/journal.pone.0312241)
Supplement: S1 Table — cNSN: prematurely condensed non-surrounded nucleolus, COC: cumulus–oocyte complexes, cSN-1: prematurely condensed surrounded nucleolus, NSN: non-surrounded nucleolus, pNSN: partly non-surrounded nucleolus, pSN-1: partly surrounded nucleolus, SN-1: surrounded nucleolus, SN-2: aggregated. a–e: There are significant differences between items with different letters in the same column (P < 0.05). Each treatment was replicated 3–4 times, and each replicate included approximately 20 COCs. (DOCX) [file pone.0312241.s001.docx]

**S1 Table. Maturation ability of rat oocytes with different chromatin configurations.** cNSN: prematurely condensed non-surrounded nucleolus, COC: cumulus–oocyte complexes, cSN-1: prematurely condensed surrounded nucleolus, NSN: non-surrounded nucleolus, pNSN: partly non-surrounded nucleolus, pSN-1: partly surrounded nucleolus, SN-1: surrounded nucleolus, SN-2: aggregated. ^a–e^: There are significant differences between items with different letters in the same column (P < 0.05). Each treatment was replicated 3–4 times, and each replicate included approximately 20 COCs.

| Chromatin configuration | Number of mature oocytes | Total number of oocytes | Mature oocyte ratio (%) |
| --- | --- | --- | --- |
| NSN | 0 | 82 | 0.00 ± 0.00^a^ |
| cNSN | 0 | 74 | 0.00 ± 0.00^a^ |
| pNSN | 0 | 92 | 0.00 ± 0.00^a^ |
| pSN-1 | 26 | 66 | 28.94 ± 1.90^b^ |
| SN-1 | 112 | 208 | 53.62 ± 0.86^c^ |
| cSN-1 | 170 | 220 | 77.16 ± 0.55^d^ |
| SN-2 | 158 | 168 | 94.22 ± 2.12^e^ |
