# Supplementary material for: Relationship between chromatin configuration and maturation ability of rat oocytes in vitro and in vivo
Source: PLoS One. 2025 Feb 13;20(2):e0312241. doi: 10.1371/journal.pone.0312241 (PMC11825056; doi:10.1371/journal.pone.0312241)
Supplement: S2 Table — GVBD: germinal vesicle breakdown, IVM: in vitro maturation. All other abbreviations are as listed in Table 1. a–q: There are significant differences between items with different letters in the same column (P < 0.05). Each treatment was replicated 3–4 times, and each replicate included approximately 15 COCs. (DOCX) [file pone.0312241.s002.docx]

**S2 Table. Changes in the chromatin configuration during IVM of rat oocytes with the NSN configuration.** GVBD: germinal vesicle breakdown, IVM: in vitro maturation. All other abbreviations are as listed in Table 1. ^a–q^: There are significant differences between items with different letters in the same column (P < 0.05). Each treatment was replicated 3–4 times, and each replicate included approximately 15 COCs.

| Culture time  (h) | Number of oocytes | Proportion of oocytes with each chromatin configuration (%) | | | | | | | |
| --- | --- | --- | --- | --- | --- | --- | --- | --- | --- |
|  |  | NSN | cNSN | pNSN | pSN-1 | SN-1 | cSN-1 | SN-2 | GVBD |
| 0.5 | 39 | 62.05 ± 1.07^a^ | 37.95 ± 1.07^a^ | 0.00 ± 0.00^a^ | 0.00 ± 0.00^a^ | 0.00 ± 0.00^a^ | 0.00 ± 0.00^a^ | 0.00 ± 0.00^a^ | 0.00 ± 0.00^a^ |
| 1 | 94 | 22.34 ± 1.35^b^ | 59.36 ± 2.68^b^ | 18.30 ± 1.95^d^ | 0.00 ± 0.00^a^ | 0.00 ± 0.00^a^ | 0.00 ± 0.00^a^ | 0.00 ± 0.00^a^ | 0.00 ± 0.00^a^ |
| 1.5 | 62 | 12.72 ± 1.13^c^ | 64.70 ±1.03^c^ | 22.58 ± 1.91^e^ | 0.00 ± 0.00^a^ | 0.00 ± 0.00^a^ | 0.00 ± 0.00^a^ | 0.00 ± 0.00^a^ | 0.00 ± 0.00^a^ |
| 2 | 68 | 0.00 ± 0.00^d^ | 30.86 ± 0.53^d^ | 50.38 ± 2.56^h^ | 15.96 ± 1.68^c^ | 2.80 ± 1.41^a^ | 0.00 ± 0.00^a^ | 0.00 ± 0.00^a^ | 0.00 ± 0.00^a^ |
| 2.5 | 56 | 0.00 ± 0.00^d^ | 10.75 ± 0.37^e^ | 46.29 ±1.85^g^ | 21.48 ± 0.74^de^ | 21.48 ± 0.47^d^ | 0.00 ± 0.00^a^ | 0.00 ± 0.00^a^ | 0.00 ± 0.00^a^ |
| 3  3.5  4  4.5  5  5.5  6  6.5  7  7.5  8  8.5  9  9.5  10  10.5  11  11.5  12  12.5  13  13.5  14  14.5  15  15.5  16 | 40  39  41  62  79  47  77  69  55  63  52  57  41  71  41  40  43  40  52  59  43  43  54  49  61  57  31 | 0.00 ± 0.00^d^  0.00 ± 0.00^d^  0.00 ± 0.00^d^  0.00 ± 0.00^d^  0.00 ± 0.00^d^  0.00 ± 0.00^d^  0.00 ± 0.00^d^  0.00 ± 0.00^d^  0.00 ± 0.00^d^  0.00 ± 0.00^d^  0.00 ± 0.00^d^  0.00 ± 0.00^d^  0.00 ± 0.00^d^  0.00 ± 0.00^d^  0.00 ± 0.00^d^  0.00 ± 0.00^d^  0.00 ± 0.00^d^  0.00 ± 0.00^d^  0.00 ± 0.00^d^  0.00 ± 0.00^d^  0.00 ± 0.00^d^  0.00 ± 0.00^d^  0.00 ± 0.00^d^  0.00 ± 0.00^d^  0.00 ± 0.00^d^  0.00 ± 0.00^d^  0.00 ± 0.00^d^ | 0.00 ± 0.00^f^  0.00 ± 0.00^f^  0.00 ± 0.00^f^  0.00 ± 0.00^f^  0.00 ± 0.00^f^  0.00 ± 0.00^f^  0.00 ± 0.00^f^  0.00 ± 0.00^f^  0.00 ± 0.00^f^  0.00 ± 0.00^f^  0.00 ± 0.00^f^  0.00 ± 0.00^f^  0.00 ± 0.00^f^  0.00 ± 0.00^f^  0.00 ± 0.00^f^  0.00 ± 0.00^f^  0.00 ± 0.00^f^  0.00 ± 0.00^f^  0.00 ± 0.00^f^  0.00 ± 0.00^f^  0.00 ± 0.00^f^  0.00 ± 0.00^f^  0.00 ± 0.00^f^  0.00 ± 0.00^f^  0.00 ± 0.00^f^  0.00 ± 0.00^f^  0.00 ± 0.00^f^ | 37.45 ± 0.60^f^  20.35 ± 1.08^de^  15.11 ± 1.86^c^  9.70 ± 0.30^b^  7.50 ± 0.18^b^  0.00 ± 0.00^a^  0.00 ± 0.00^a^  0.00 ± 0.00^a^  0.00 ± 0.00^a^  0.00 ± 0.00^a^  0.00 ± 0.00^a^  0.00 ± 0.00^a^  0.00 ± 0.00^a^  0.00 ± 0.00^a^  0.00 ± 0.00^a^  0.00 ± 0.00^a^  0.00 ± 0.00^a^  0.00 ± 0.00^a^  0.00 ± 0.00^a^  0.00 ± 0.00^a^  0.00 ± 0.00^a^  0.00 ± 0.00^a^  0.00 ± 0.00^a^  0.00 ± 0.00^a^  0.00 ± 0.00^a^  0.00 ± 0.00^a^  0.00 ± 0.00^a^ | 29.28 ± 4.28^gh^  35.93 ± 0.22^ij^  38.67 ± 1.40^jk^  41.82 ± 1.82^kl^  42.07 ± 0.54^kl^  47.22 ± 1.47^m^  46.61 ± 0.91^m^  46.35 ± 0.70^m^  43.54 ± 1.19^lm^  41.40 ± 1.83^kl^  32.64 ± 0.69^hi^  28.04 ± 0.26^fg^  26.86 ± 0.20^fg^  25.25 ± 1.28^ef^  24.36 ± 0.64^ef^  22.56 ± 2.63^de^  20.65 ± 2.01^d^  15.02 ± 0.36^c^  12.86 ± 1.43^c^  5.14 ± 0.39^b^  0.00 ± 0.00^a^  0.00 ± 0.00^a^  0.00 ± 0.00^a^  0.00 ± 0.00^a^  0.00 ± 0.00^a^  0.00 ± 0.00^a^  0.00 ± 0.00^a^ | 33.27 ± 4.18^f^  43.72 ± 0.86^gh^  46.22 ± 0.47^hij^  48.48 ± 1.52^ijk^  50.43 ± 0.43^jklm^  52.78 ± 1.47^klmn^  53.39 ± 0.91^lmno^  53.65 ± 0.70^lmno^  56.46 ± 1.19^nop^  58.60 ± 1.84^p^  67.36 ± 0.69^a^  68.26 ± 1.59^q^  68.69 ± 2.02^q^  57.77 ± 0.74^op^  56.14 ± 1.29^nop^  54.88 ± 0.34^mnop^  53.35 ± 3.69^lmno^  50.00 ± 2.22^jkl^  45.24 ± 2.38^hi^  40.70 ± 0.36^g^  39..49 ± 0.51^g^  28.11 ± 1.68^e^  24.12 ± 0.45^de^  20.47 ± 0.48^cd^  16.57 ± 0.66^c^  11.94 ± 1.00^b^  9.70 ± 0.30^b^ | 0.00 ± 0.00^a^  0.00 ± 0.00^a^  0.00 ± 0.00^a^  0.00 ± 0.00^a^  0.00 ± 0.00^a^  0.00 ± 0.00^a^  0.00 ± 0.00^a^  0.00 ± 0.00^a^  0.00 ± 0.00^a^  0.00 ± 0.00^a^  0.00 ± 0.00^a^  3.70 ± 1.85^ab^  4.45 ± 2.22^b^  16.98 ± 0.83^c^  19.50 ± 1.89^cd^  22.56 ± 2.63^d^  26.00 ± 2.52^e^  34.98 ± 2.52^e^  41.90 ± 0.95^g^  49.02 ± 0.98^h^  53.50 ± 0.17^i^  55.75 ± 0.98^ij^  57.40 ± 0.75^jk^  59.05 ± 0.95^jkl^  61.77 ± 1.92^l^  60.83 ± 0.83^kl^  58.18 ± 1.82^jk^ | 0.00 ± 0.00^a^  0.00 ± 0.00^a^  0.00 ± 0.00^a^  0.00 ± 0.00^a^  0.00 ± 0.00^a^  0.00 ± 0.00^a^  0.00 ± 0.00^a^  0.00 ± 0.00^a^  0.00 ± 0.00^a^  0.00 ± 0.00^a^  0.00 ± 0.00^a^  0.00 ± 0.00^a^  0.00 ± 0.00^a^  0.00 ± 0.00^a^  0.00 ± 0.00^a^  0.00 ± 0.00^a^  0.00 ± 0.00^a^  0.00 ±0.00^a^  0.00 ± 0.00^a^  5.14 ± 0.39^b^  7.01 ± 0.34^c^  16.14 ± 1.34^d^  18.48 ± 0.43^e^  20.48 ± 0.48^f^  21.66 ± 1.27^f^  27.23 ± 1.47^g^  32.12 ± 2.12^h^ | 0.00 ± 0.00^a^  0.00 ± 0.00^a^  0.00 ± 0.00^a^  0.00 ± 0.00^a^  0.00 ± 0.00^a^  0.00 ± 0.00^a^  0.00 ± 0.00^a^  0.00 ± 0.00^a^  0.00 ± 0.00^a^  0.00 ± 0.00^a^  0.00 ± 0.00^a^  0.00 ± 0.00^a^  0.00 ± 0.00^a^  0.00 ± 0.00^a^  0.00 ± 0.00^a^  0.00 ± 0.00^a^  0.00 ± 0.00^a^  0.00 ± 0.00^a^  0.00 ± 0.00^a^  0.00 ± 0.00^a^  0.00 ± 0.00^a^  0.00 ± 0.00^a^  0.00 ± 0.00^a^  0.00 ± 0.00^a^  0.00 ± 0.00^a^  0.00 ± 0.00^a^  0.00 ± 0.00^a^ |
