# Supplementary material for: Relationship between chromatin configuration and maturation ability of rat oocytes in vitro and in vivo
Source: PLoS One. 2025 Feb 13;20(2):e0312241. doi: 10.1371/journal.pone.0312241 (PMC11825056; doi:10.1371/journal.pone.0312241)
Supplement: S3 Table — GVBD: germinal vesicle breakdown, IVM: in vitro maturation. All other abbreviations are as listed in Table 1. a–q: There are significant differences between items with different letters in the same column (P < 0.05). Each treatment was replicated 3–4 times, and each replicate included approximately 15 COCs. (DOCX) [file pone.0312241.s003.docx]

**S3 Table.** **Changes in the chromatin configuration during IVM of rat oocytes with the cNSN configuration.** GVBD: germinal vesicle breakdown, IVM: in vitro maturation. All other abbreviations are as listed in Table 1. ^a–q^: There are significant differences between items with different letters in the same column (P < 0.05). Each treatment was replicated 3–4 times, and each replicate included approximately 15 COCs.

| Culture time (h) | Number of oocytes | | Proportion of oocytes with each chromatin configuration (%) | | | | | | |
| --- | --- | --- | --- | --- | --- | --- | --- | --- | --- |
|  |  |  | cNSN | pNSN | pSN-1 | SN-1 | cSN-1 | SN-2 | GVBD |
| 0.5 | | 51 | 50.23 ± 2.43^a^ | 49.77 ± 2.43^a^ | 0.00 ± 0.00^a^ | 0.00 ± 0.00^a^ | 0.00 ± 0.00^a^ | 0.00 ± 0.00^a^ | 0.00 ± 0.00^a^ |
| 1 | | 61 | 24.27 ± 3.33^b^ | 50.90 ± 1.90^a^ | 16.55 ± 0.97^d^ | 8.28 ± 0.49^b^ | 0.00 ± 0.00^a^ | 0.00 ± 0.00^a^ | 0.00 ± 0.00^a^ |
| 1.5 | | 95 | 15.71 ± 1.19^c^ | 57.94 ± 1.04^b^ | 16.83 ± 0.16^d^ | 9.52 ± 0.48^b^ | 0.00 ±0.00^a^ | 0.00 ± 0.00^a^ | 0.00 ± 0.00^a^ |
| 2 | | 58 | 0.00 ± 0.00^d^ | 52.24 ± 1.31^a^ | 20.25 ± 1.07^e^ | 27.51 ± 0.89^ef^ | 0.00 ± 0.00^a^ | 0.00 ± 0.00^a^ | 0.00 ± 0.00^a^ |
| 2.5 | | 66 | 0.00 ± 0.00^d^ | 40.43 ± 2.20^c^ | 30.40 ± 0.59^fg^ | 29.17 ± 1.68^fg^ | 0.00 ± 0.00^a^ | 0.00 ± 0.00^a^ | 0.00 ± 0.00^a^ |
| 3 | | 50 | 0.00 ± 0.00^d^ | 15.63 ± 2.72^d^ | 34.14 ± 1.76^hi^ | 44.17 ± 1.33^j^ | 6.06 ± 0.42^b^ | 0.00 ± 0.00^a^ | 0.00 ± 0.00^a^ |
| 3.5 | | 52 | 0.00 ± 0.00^d^ | 11.17 ± 1.76^e^ | 35.04 ± 1.71^i^ | 46.23 ± 0.24^j^ | 7.56 ± 0.48^b^ | 0.00 ± 0.00^a^ | 0.00 ± 0.00^a^ |
| 4 | | 74 | 0.00 ± 0.00^d^ | 4.07 ± 0.15^f^ | 32.35 ± 1.22^gh^ | 50.11 ± 1.98^k^ | 13.47 ± 1.00^cd^ | 0.00 ± 0.00^a^ | 0.00 ± 0.00^a^ |
| 4.5 | | 75 | 0.00 ± 0.00^d^ | 0.00 ± 0.00^g^ | 32.03 ± 0.74^g^ | 51.95 ± 1.11^kl^ | 16.02 ± 0.37^de^ | 0.00 ± 0.00^a^ | 0.00 ± 0.00^a^ |
| 5 | | 48 | 0.00 ± 0.00^d^ | 0.00 ± 0.00^g^ | 29.11 ± 1.33^f^ | 54.31 ± 2.97^lm^ | 16.58 ± 1.65^def^ | 0.00 ± 0.00^a^ | 0.00 ± 0.00^a^ |
| 5.5 | | 56 | 0.00 ± 0.00^d^ | 0.00 ± 0.00^g^ | 21.53 ± 1.05^e^ | 58.90 ± 0.61^no^ | 19.57 ± 1.01^fgh^ | 0.00 ± 0.00^a^ | 0.00 ± 0.00^a^ |
| 6 | | 63 | 0.00 ± 0.00^d^ | 0.00 ± 0.00^g^ | 17.41 ± 0.94^d^ | 61.98 ± 0.66^op^ | 20.61 ± 0.81^gh^ | 0.00 ± 0.00^a^ | 0.00 ± 0.00^a^ |
| 6.5 | | 47 | 0.00 ± 0.00^d^ | 0.00 ± 0.00^g^ | 12.90 ± 0.94^c^ | 65.88 ± 0.79^q^ | 21.22 ± 0.65^h^ | 0.00 ± 0.00^a^ | 0.00 ± 0.00^a^ |
| 7 | | 40 | 0.00 ± 0.00^d^ | 0.00 ± 0.00^g^ | 7.56 ± 0.48^b^ | 67.52 ± 0.85^q^ | 24.92 ± 1.04^i^ | 0.00 ± 0.00^a^ | 0.00 ± 0.00^a^ |
| 7.5 | | 34 | 0.00 ± 0.00^d^ | 0.00 ± 0.00^g^ | 0.00 ± 0.00^a^ | 65.06 ± 2.54^pq^ | 34.94 ± 2.54^k^ | 0.00 ± 0.00^a^ | 0.00 ± 0.00^a^ |
| 8 | | 41 | 0.00 ± 0.00^d^ | 0.00 ± 0.00^g^ | 0.00 ± 0.00^a^ | 58.71 ± 1.54^no^ | 41.29 ± 1.54^l^ | 0.00 ± 0.00^a^ | 0.00 ± 0.00^a^ |
| 8.5 | | 34 | 0.00 ± 0.00^d^ | 0.00 ± 0.00^g^ | 0.00 ± 0.00^a^ | 56.13 ± 1.94^mn^ | 43.87 ± 1.94^l^ | 0.00 ± 0.00^a^ | 0.00 ± 0.00^a^ |
| 9 | | 57 | 0.00 ± 0.00^d^ | 0.00 ± 0.00^g^ | 0.00 ± 0.00^a^ | 45.32 ± 1.23^j^ | 54.68 ± 1.23^m^ | 0.00 ± 0.00^a^ | 0.00 ± 0.00^a^ |
| 9.5 | | 73 | 0.00 ± 0.00^d^ | 0.00 ± 0.00^g^ | 0.00 ± 0.00^a^ | 43.84 ± 0.32^j^ | 56.16 ± 0.32^m^ | 0.00 ± 0.00^a^ | 0.00 ± 0.00^a^ |
| 10 | | 63 | 0.00 ± 0.00^d^ | 0.00 ± 0.00^g^ | 0.00 ± 0.00^a^ | 38.14 ± 0.32^i^ | 61.86 ± 0.32^n^ | 0.00 ± 0.00^a^ | 0.00 ± 0.00^a^ |
| 10.5 | | 49 | 0.00 ± 0.00^d^ | 0.00 ± 0.00^g^ | 0.00 ± 0.00^a^ | 34.90 ± 0.88^h^ | 65.10 ± 0.88^o^ | 0.00 ± 0.00^a^ | 0.00 ± 0.00^a^ |
| 11 | | 45 | 0.00 ± 0.00^d^ | 0.00 ± 0.00^g^ | 0.00 ± 0.00^a^ | 31.44 ± 1.13^g^ | 68.56 ± 1.13^p^ | 0.00 ± 0.00^a^ | 0.00 ± 0.00^a^ |
| 11.5 | | 55 | 0.00 ± 0.00^d^ | 0.00 ± 0.00^g^ | 0.00 ± 0.00^a^ | 25.72 ± 1.02^e^ | 74.28 ± 1.02^q^ | 0.00 ± 0.00^a^ | 0.00 ± 0.00^a^ |
| 12 | | 45 | 0.00 ± 0.00^d^ | 0.00 ± 0.00^g^ | 0.00 ± 0.00^a^ | 20.31 ± 1.37^d^ | 68.56 ± 1.13^p^ | 11.13 ± 0.36^b^ | 0.00 ± 0.00^a^ |
| 12.5 | | 38 | 0.00 ± 0.00^d^ | 0.00 ± 0.00^g^ | 0.00 ± 0.00^a^ | 13.10 ± 0.60^c^ | 63.10 ± 0.60^no^ | 23.80 ± 1.19^c^ | 0.00 ± 0.00^a^ |
| 13 | | 57 | 0.00 ± 0.00^d^ | 0.00 ± 0.00^g^ | 0.00 ± 0.00^a^ | 8.70 ± 0.22^b^ | 42.02 ± 0.76^l^ | 49.28 ± 0.72^d^ | 0.00 ± 0.00^a^ |
| 13.5 | | 53 | 0.00 ± 0.00^d^ | 0.00 ± 0.00^g^ | 0.00 ± 0.00^a^ | 0.00 ± 0.00^a^ | 35.93 ± 1.39^k^ | 64.07 ± 1.39^e^ | 0.00 ± 0.00^a^ |
| 14 | | 50 | 0.00 ± 0.00^d^ | 0.00 ± 0.00^g^ | 0.00 ± 0.00^a^ | 0.00 ± 0.00^a^ | 28.30 ± 1.07^j^ | 71.70 ± 1.07^f^ | 0.00 ± 0.00^a^ |
| 14.5 | | 45 | 0.00 ± 0.00^d^ | 0.00 ± 0.00^g^ | 0.00 ± 0.00^a^ | 0.00 ± 0.00^a^ | 20.31 ± 1.37^gh^ | 79.69 ± 1.37^g^ | 0.00 ± 0.00^a^ |
| 15 | | 79 | 0.00 ± 0.00^d^ | 0.00 ± 0.00^g^ | 0.00 ± 0.00^a^ | 0.00 ± 0.00^a^ | 17.87 ± 0.62^efg^ | 82.13 ± 0.62^h^ | 0.00 ± 0.00^a^ |
| 15.5 | | 90 | 0.00 ± 0.00^d^ | 0.00 ± 0.00^g^ | 0.00 ± 0.00^a^ | 0.00 ± 0.00^a^ | 14.66 ± 0.58^cd^ | 85.34 ± 0.58^i^ | 0.00 ± 0.00^a^ |
| 16 | | 33 | 0.00 ± 0.00^d^ | 0.00 ± 0.00^g^ | 0.00 ± 0.00^a^ | 0.00 ± 0.00^a^ | 12.04 ± 0.46^c^ | 87.96 ± 0.46^j^ | 0.00 ± 0.00^a^ |
