# Supplementary material for: Relationship between chromatin configuration and maturation ability of rat oocytes in vitro and in vivo
Source: PLoS One. 2025 Feb 13;20(2):e0312241. doi: 10.1371/journal.pone.0312241 (PMC11825056; doi:10.1371/journal.pone.0312241)
Supplement: S4 Table — GVBD: germinal vesicle breakdown, IVM: in vitro maturation. All other abbreviations are as listed in Table 1. a–t: There are significant differences between items with different letters in the same column (P < 0.05). Each treatment was replicated 3–4 times, and each replicate included approximately 15 COCs. (DOCX) [file pone.0312241.s004.docx]

**S4 Table. Changes in the chromatin configuration during IVM of rat oocytes with the pNSN configuration.** GVBD: germinal vesicle breakdown, IVM: in vitro maturation. All other abbreviations are as listed in Table 1. ^a–t^: There are significant differences between items with different letters in the same column (P < 0.05). Each treatment was replicated 3–4 times, and each replicate included approximately 15 COCs.

| Culture time (h) | Number of oocytes | Proportion of oocytes with each chromatin configuration (%) | | | | | |
| --- | --- | --- | --- | --- | --- | --- | --- |
|  |  | pNSN | pSN-1 | SN-1 | cSN-1 | SN-2 | GVBD |
| 0.5 | 59 | 57.57 ± 1.29^a^ | 42.43 ± 1.29^g^ | 0.00± 0.00^a^ | 0.00 ± 0.00^a^ | 0.00 ± 0.00^a^ | 0.00 ± 0.00^a^ |
| 1 | 57 | 22.87 ± 2.47^b^ | 45.65 ± 6.65^h^ | 31.48 ± 1.85^e^ | 0.00 ± 0.00^a^ | 0.00 ± 0.00^a^ | 0.00 ± 0.00^a^ |
| 1.5 | 80 | 8.48 ± 2.13^c^ | 52.16 ± 1.14^i^ | 39.36 ± 1.40^g^ | 0.00 ± 0.00^a^ | 0.00 ± 0.00^a^ | 0.00 ± 0.00^a^ |
| 2 | 51 | 0.00 ± 0.00^d^ | 47.63 ± 0.20^h^ | 52.97 ± 0.20^ij^ | 0.00 ± 0.00^a^ | 0.00 ± 0.00^a^ | 0.00 ± 0.00^a^ |
| 2.5 | 74 | 0.00 ± 0.00^d^ | 45.65 ± 0.76^h^ | 54.35 ± 0.76^j^ | 0.00 ± 0.00^a^ | 0.00 ± 0.00^a^ | 0.00 ± 0.00^a^ |
| 3 | 47 | 0.00 ± 0.00^d^ | 38.20 ± 1.29^f^ | 51.11 ± 1.11^i^ | 10.69 ± 2.21^b^ | 0.00 ± 0.00^a^ | 0.00 ± 0.00^a^ |
| 3.5 | 58 | 0.00 ± 0.00^d^ | 34.44 ± 0.56^e^ | 44.82 ± 0.19^h^ | 20.74 ± 0.74^d^ | 0.00 ± 0.00^a^ | 0.00 ± 0.00^a^ |
| 4 | 43 | 0.00 ± 0.00^d^ | 27.86 ± 1.49^d^ | 41.51 ± 0.83^g^ | 30.63 ± 1.41^fg^ | 0.00 ± 0.00^a^ | 0.00 ± 0.00^a^ |
| 4.5 | 41 | 0.00 ± 0.00^d^ | 23.91 ± 3.62^c^ | 38.97 ± 1.53^g^ | 34.34 ± 1.01^gh^ | 2.78 ± 2.78^a^ | 0.00 ± 0.00^a^ |
| 5 | 43 | 0.00 ± 0.00^d^ | 13.70 ± 3.16^b^ | 35.56 ± 2.22^f^ | 41.48 ± 1.48^i^ | 9.26 ± 1.33^b^ | 0.00 ± 0.00^a^ |
| 5.5 | 43 | 0.00 ± 0.00^d^ | 2.38 ± 2.38^a^ | 32.54 ± 2.09^e^ | 51.11 ± 4.27^k^ | 13.97 ± 0.32^c^ | 0.00 ± 0.00^a^ |
| 6 | 56 | 0.00 ± 0.00^d^ | 0.00 ± 0.00^a^ | 28.29 ± 0.87^d^ | 54.11 ± 1.58^kl^ | 17.60 ± 1.71^d^ | 0.00 ± 0.00^a^ |
| 6.5 | 48 | 0.00 ± 0.00^d^ | 0.00 ± 0.00^a^ | 18.89 ± 1.11^c^ | 62.22 ± 2.22^no^ | 18.89 ± 1.11^d^ | 0.00 ± 0.00^a^ |
| 7 | 60 | 0.00 ± 0.00^d^ | 0.00 ± 0.00^a^ | 10.43 ± 1.43^b^ | 66.14 ± 1.96^opq^ | 23.43 ± 0.82^e^ | 0.00 ± 0.00^a^ |
| 7.5 | 58 | 0.00 ± 0.00^d^ | 0.00 ± 0.00^a^ | 0.00 ± 0.00^a^ | 75.93 ± 0.93^s^ | 24.07 ± 0.93^e^ | 0.00 ± 0.00^a^ |
| 8 | 57 | 0.00 ± 0.00^d^ | 0.00 ± 0.00^a^ | 0.00 ± 0.00^a^ | 72.40 ± 1.73^rs^ | 27.60 ± 1.73^f^ | 0.00 ± 0.00^a^ |
| 8.5 | 70 | 0.00 ± 0.00^d^ | 0.00 ± 0.00^a^ | 0.00 ± 0.00^a^ | 70.10 ± 1.39^qr^ | 29.90 ± 1.39^fg^ | 0.00 ± 0.00^a^ |
| 9 | 66 | 0.00 ± 0.00^d^ | 0.00 ± 0.00^a^ | 0.00 ± 0.00^a^ | 68.28 ± 1.79^pq^ | 31.72 ± 1.79^gh^ | 0.00 ± 0.00^a^ |
| 9.5 | 68 | 0.00 ± 0.00^d^ | 0.00 ± 0.00^a^ | 0.00 ± 0.00^a^ | 67.98 ± 1.31^pq^ | 32.02 ± 1.31^gh^ | 0.00 ± 0.00^a^ |
| 10 | 70 | 0.00 ± 0.00^d^ | 0.00 ± 0.00^a^ | 0.00 ± 0.00^a^ | 65.50 ± 1.17^op^ | 34.50 ± 1.17^hi^ | 0.00 ± 0.00^a^ |
| 10.5 | 74 | 0.00 ± 0.00^d^ | 0.00 ± 0.00^a^ | 0.00 ± 0.00^a^ | 64.87 ± 0.44^op^ | 35.13 ± 0.44^ij^ | 0.00 ± 0.00^a^ |
| 11 | 63 | 0.00 ± 0.00^d^ | 0.00 ± 0.00^a^ | 0.00 ± 0.00^a^ | 61.98 ± 0.66^no^ | 38.02 ± 0.66^jk^ | 0.00 ± 0.00^a^ |
| 11.5 | 120 | 0.00 ± 0.00^d^ | 0.00 ± 0.00^a^ | 0.00 ± 0.00^a^ | 59.98 ± 0.58^mn^ | 40.02 ± 0.58^kl^ | 0.00 ± 0.00^a^ |
| 12 | 87 | 0.00 ± 0.00^d^ | 0.00 ± 0.00^a^ | 0.00 ± 0.00^a^ | 57.17 ± 0.94^lm^ | 42.83 ± 0.94^l^ | 0.00 ± 0.00^a^ |
| 12.5 | 59 | 0.00 ± 0.00^d^ | 0.00 ± 0.00^a^ | 0.00 ± 0.00^a^ | 52.54 ± 1.44^k^ | 47.46 ± 1.44^m^ | 0.00 ± 0.00^a^ |
| 13 | 61 | 0.00 ± 0.00^d^ | 0.00 ± 0.00^a^ | 0.00 ± 0.00^a^ | 45.94 ± 0.73^j^ | 54.06 ± 0.73^n^ | 0.00 ± 0.00^a^ |
| 13.5 | 63 | 0.00 ± 0.00^d^ | 0.00 ± 0.00^a^ | 0.00 ± 0.00^a^ | 39.53 ± 1.07^i^ | 60.47 ± 1.07^o^ | 0.00 ± 0.00^a^ |
| 14 | 66 | 0.00 ± 0.00^d^ | 0.00 ± 0.00^a^ | 0.00 ± 0.00^a^ | 34.82 ± 0.87^h^ | 65.18 ± 0.87^p^ | 0.00 ± 0.00^a^ |
| 14.5 | 63 | 0.00 ± 0.00^d^ | 0.00 ± 0.00^a^ | 0.00 ± 0.00^a^ | 27.67 ± 0.61^ef^ | 72.93 ± 0.61^q^ | 0.00 ± 0.00^a^ |
| 15 | 50 | 0.00 ± 0.00^d^ | 0.00 ± 0.00^a^ | 0.00 ± 0.00^a^ | 23.68 ± 0.81^de^ | 76.32 ± 0.81^r^ | 0.00 ± 0.00^a^ |
| 15.5 | 84 | 0.00 ± 0.00^d^ | 0.00 ± 0.00^a^ | 0.00 ± 0.00^a^ | 15.45 ± 0.91^c^ | 84.55 ± 0.91^s^ | 0.00 ± 0.00^a^ |
| 16 | 80 | 0.00 ± 0.00^d^ | 0.00 ± 0.00^a^ | 0.00 ± 0.00^a^ | 9.87 ± 0.42^b^ | 90.13 ± 0.42^t^ | 0.00 ± 0.00^a^ |
