# Supplementary material for: Relationship between chromatin configuration and maturation ability of rat oocytes in vitro and in vivo
Source: PLoS One. 2025 Feb 13;20(2):e0312241. doi: 10.1371/journal.pone.0312241 (PMC11825056; doi:10.1371/journal.pone.0312241)
Supplement: S5 Table — GVBD: germinal vesicle breakdown, IVM: in vitro maturation. All other abbreviations are as listed in Table 1. a–r: There are significant differences between items with different letters in the same column (P < 0.05). Each treatment was replicated 3–4 times, and each replicate included approximately 15 COCs. (DOCX) [file pone.0312241.s005.docx]

**S5 Table. Changes in the chromatin configuration during IVM of rat oocytes with the pSN-1 configuration.** GVBD: germinal vesicle breakdown, IVM: in vitro maturation. All other abbreviations are as listed in Table 1. ^a–r^: There are significant differences between items with different letters in the same column (P < 0.05). Each treatment was replicated 3–4 times, and each replicate included approximately 15 COCs.

| Culture time (h) | Number of oocytes | Proportion of oocytes with each chromatin configuration (%) | | | | |
| --- | --- | --- | --- | --- | --- | --- |
|  |  | pSN-1 | SN-1 | cSN-1 | SN-2 | GVBD |
| 0.5 | 68 | 49.83 ± 2.07^a^ | 50.17 ± 2.07^a^ | 0.00 ± 0.00^a^ | 0.00 ± 0.00^a^ | 0.00 ± 0.00^a^ |
| 1 | 76 | 17.53 ± 0.85^b^ | 81.19 ± 2.14^b^ | 1.28 ± 1.28^ab^ | 0.00 ± 0.00^a^ | 0.00 ± 0.00^a^ |
| 1.5 | 48 | 0.00 ± 0.00^c^ | 79.26 ± 0.74^c^ | 20.74 ± 0.74^ef^ | 0.00 ± 0.00^a^ | 0.00 ± 0.00^a^ |
| 2 | 45 | 0.00 ± 0.00^c^ | 48.85 ± 2.26^a^ | 51.15 ± 2.26^k^ | 0.00 ± 0.00^a^ | 0.00 ± 0.00^a^ |
| 2.5 | 66 | 0.00 ± 0.00^c^ | 41.90 ± 2.21^d^ | 58.10 ± 2.21^l^ | 0.00 ± 0.00^a^ | 0.00 ± 0.00^a^ |
| 3 | 54 | 0.00 ± 0.00^c^ | 18.52 ± 1.85^e^ | 81.48 ± 1.85^q^ | 0.00 ± 0.00^a^ | 0.00 ± 0.00^a^ |
| 3.5 | 70 | 0.00 ± 0.00^c^ | 11.36 ± 2.66^f^ | 82.83 ± 0.50^q^ | 5.81 ± 2.91^b^ | 0.00 ± 0.00^a^ |
| 4 | 60 | 0.00 ± 0.00^c^ | 2.57 ± 2.56^g^ | 87.00 ± 1.26^r^ | 10.43 ± 1.43^c^ | 0.00 ± 0.00^a^ |
| 4.5 | 74 | 0.00 ± 0.00^c^ | 0.00 ± 0.00^g^ | 76.11 ± 2.00^p^ | 23.89 ± 2.00^d^ | 0.00 ± 0.00^a^ |
| 5 | 59 | 0.00 ± 0.00^c^ | 0.00 ± 0.00^g^ | 72.60 ± 1.29^op^ | 27.40 ± 1.29^de^ | 0.00 ± 0.00^a^ |
| 5.5 | 71 | 0.00 ± 0.00^c^ | 0.00 ± 0.00^g^ | 71.93 ± 2.33^no^ | 28.07 ± 2.32^ef^ | 0.00 ± 0.00^a^ |
| 6 | 57 | 0.00 ± 0.00^c^ | 0.00 ± 0.00^g^ | 70.23 ± 0.92^no^ | 29.77 ± 0.92^ef^ | 0.00 ± 0.00^a^ |
| 6.5 | 60 | 0.00 ± 0.00^c^ | 0.00 ± 0.00^g^ | 68.29 ± 0.81^n^ | 31.71 ± 0.81^f^ | 0.00 ± 0.00^a^ |
| 7 | 86 | 0.00 ± 0.00^c^ | 0.00 ± 0.00^g^ | 62.74 ± 0.47^m^ | 37.26 ± 0.47^g^ | 0.00 ± 0.00^a^ |
| 7.5 | 63 | 0.00 ± 0.00^c^ | 0.00 ± 0.00^g^ | 60.39 ± 0.79^lm^ | 39.61 ± 0.79^gh^ | 0.00 ± 0.00^a^ |
| 8 | 56 | 0.00 ± 0.00^c^ | 0.00 ± 0.00^g^ | 58.59 ± 2.59^l^ | 41.41 ± 2.59^h^ | 0.00 ± 0.00^a^ |
| 8.5 | 58 | 0.00 ± 0.00^c^ | 0.00 ± 0.00^g^ | 50.44 ± 1.55^k^ | 49.56 ± 1.55^i^ | 0.00 ± 0.00^a^ |
| 9 | 64 | 0.00 ± 0.00^c^ | 0.00 ± 0.00^g^ | 45.50 ± 0.79^j^ | 54.50 ± 0.79^j^ | 0.00 ± 0.00^a^ |
| 9.5 | 75 | 0.00 ± 0.00^c^ | 0.00 ± 0.00^g^ | 41.70 ± 1.49^i^ | 58.30 ± 1.50^k^ | 0.00 ± 0.00^a^ |
| 10 | 59 | 0.00 ± 0.00^c^ | 0.00 ± 0.00^g^ | 39.12 ± 0.46^i^ | 60.88 ± 0.46^kl^ | 0.00 ± 0.00^a^ |
| 10.5 | 64 | 0.00 ± 0.00^c^ | 0.00 ± 0.00^g^ | 32.83 ± 0.50^h^ | 67.17 ± 0.50^mn^ | 0.00 ± 0.00^a^ |
| 11 | 50 | 0.00 ± 0.00^c^ | 0.00 ± 0.00^g^ | 29.95 ± 0.29^gh^ | 70.05 ± 0.30^no^ | 0.00 ± 0.00^a^ |
| 11.5 | 46 | 0.00 ± 0.00^c^ | 0.00 ± 0.00^g^ | 28.30 ± 1.07^g^ | 71.70 ± 1.07^op^ | 0.00 ± 0.00^a^ |
| 12 | 52 | 0.00 ± 0.00^c^ | 0.00 ± 0.00^g^ | 23.68 ± 1.86^f^ | 76.32 ± 1.86^q^ | 0.00 ± 0.00^a^ |
| 12.5 | 79 | 0.00 ± 0.00^c^ | 0.00 ± 0.00^g^ | 18.72 ± 0.69^de^ | 81.28 ± 0.69^r^ | 0.00 ± 0.00^a^ |
| 13 | 49 | 0.00 ± 0.00^c^ | 0.00 ± 0.00^g^ | 16.71 ± 1.22^d^ | 83.29 ± 1.22^r^ | 0.00 ± 0.00^a^ |
| 13.5 | 68 | 0.00 ± 0.00^c^ | 0.00 ± 0.00^g^ | 13.01 ± 1.73^c^ | 82.54 ± 1.44^r^ | 4.45 ± 0.29^b^ |
| 14 | 67 | 0.00 ± 0.00^c^ | 0.00 ± 0.00^g^ | 4.48 ± 0.14^b^ | 80.61 ± 1.27^r^ | 14.91 ± 1.29^c^ |
| 14.5 | 72 | 0.00 ± 0.00^c^ | 0.00 ± 0.00^g^ | 0.00 ± 0.00^a^ | 75.03 ± 2.14^pq^ | 24.97 ± 2.14^d^ |
| 15 | 66 | 0.00 ± 0.00^c^ | 0.00 ± 0.00^g^ | 0.00 ± 0.00^a^ | 66.69 ± 0.85^mn^ | 33.31 ± 0.85^e^ |
| 15.5 | 78 | 0.00 ± 0.00^c^ | 0.00 ± 0.00^g^ | 0.00 ± 0.00^a^ | 63.83 ± 0.68^lm^ | 36.17 ± 0.68^f^ |
| 16 | 58 | 0.00 ± 0.00^c^ | 0.00 ± 0.00^g^ | 0.00 ± 0.00^a^ | 61.89 ± 0.96^kl^ | 38.11 ± 0.96^g^ |
