# Supplementary material for: Relationship between chromatin configuration and maturation ability of rat oocytes in vitro and in vivo
Source: PLoS One. 2025 Feb 13;20(2):e0312241. doi: 10.1371/journal.pone.0312241 (PMC11825056; doi:10.1371/journal.pone.0312241)
Supplement: S6 Table — GVBD: germinal vesicle breakdown, IVM: in vitro maturation. All other abbreviations are as listed in Table 1. a–i: There are significant differences between items with different letters in the same column (P < 0.05). Each treatment was replicated 3–4 times, and each replicate included approximately 30 COCs. (DOCX) [file pone.0312241.s006.docx]

**S6 Table. Changes in the chromatin configuration during IVM of rat oocytes with the SN-1 configuration.** GVBD: germinal vesicle breakdown, IVM: in vitro maturation. All other abbreviations are as listed in Table 1. ^a–i^: There are significant differences between items with different letters in the same column (P < 0.05). Each treatment was replicated 3–4 times, and each replicate included approximately 30 COCs.

| Culture time (h) | Number of oocytes | Proportion of oocytes with each chromatin configuration (%) | | | |
| --- | --- | --- | --- | --- | --- |
|  |  | SN-1 | cSN-1 | SN-2 | GVBD |
| 0.5 | 49 | 91.86 ± 0.95^a^ | 8.14 ± 0.95^a^ | 0.00 ± 0.00^a^ | 0.00 ± 0.00^a^ |
| 1 | 69 | 86.57 ± 1.67^b^ | 13.43 ± 1.67^a^ | 0.00 ± 0.00^a^ | 0.00 ± 0.00^a^ |
| 1.5 | 75 | 79.87 ± 1.17^c^ | 20.13 ± 1.17^c^ | 0.00 ± 0.00^a^ | 0.00 ± 0.00^a^ |
| 2 | 74 | 65.50 ± 1.17^d^ | 30.80 ± 4.41^e^ | 3.70 ± 3.70^ab^ | 0.00 ± 0.00^a^ |
| 2.5 | 68 | 40.42 ± 1.57^e^ | 43.55 ± 1.76^f^ | 16.03 ± 1.14^d^ | 0.00 ± 0.00^a^ |
| 3 | 63 | 33.27 ± 4.60^f^ | 49.05 ± 3.50^fg^ | 17.68 ± 1.23^de^ | 0.00 ± 0.00^a^ |
| 3.5 | 70 | 2.38 ± 2.38^g^ | 60.72 ± 2.99^i^ | 22.88 ± 1.09^fg^ | 14.02 ± 1.61^b^ |
| 4 | 88 | 0.00 ± 0.00^g^ | 56.54 ± 1.24^hi^ | 25.27 ± 1.09^fgh^ | 18.19 ± 0.32^bc^ |
| 4.5 | 86 | 0.00 ± 0.00^g^ | 52.95 ± 1.51^gh^ | 28.59 ± 1.81^h^ | 18.46 ± 1.54^c^ |
| 5 | 84 | 0.00 ± 0.00^g^ | 50.64 ± 2.09^gh^ | 26.19 ± 0.66^gh^ | 23.17 ± 2.52^d^ |
| 5.5 | 80 | 0.00 ± 0.00^g^ | 48.15 ± 1.85^fg^ | 21.48 ± 3.29^efg^ | 30.37 ± 1.61^e^ |
| 6 | 76 | 0.00 ± 0.00^g^ | 42.94 ± 2.04^f^ | 20.87 ± 1.35^ef^ | 36.19 ± 1.61^f^ |
| 6.5 | 86 | 0.00 ± 0.00^g^ | 30.32 ± 0.45^de^ | 14.17 ± 1.21^d^ | 55.51 ± 1.66^g^ |
| 7 | 86 | 0.00 ± 0.00^g^ | 31.57 ± 3.39^e^ | 9.14 ± 0.48^c^ | 59.29 ± 3.05^g^ |
| 7.5 | 76 | 0.00 ± 0.00^g^ | 23.93 ± 1.74^cd^ | 7.97 ± 0.58^bc^ | 68.10 ± 2.32^h^ |
| 8 | 90 | 0.00 ± 0.00^g^ | 0.00 ± 0.00^b^ | 6.69 ± 0.26^bc^ | 93.31 ± 0.26^i^ |
