# Supplementary material for: Relationship between chromatin configuration and maturation ability of rat oocytes in vitro and in vivo
Source: PLoS One. 2025 Feb 13;20(2):e0312241. doi: 10.1371/journal.pone.0312241 (PMC11825056; doi:10.1371/journal.pone.0312241)
Supplement: S7 Table — GVBD: germinal vesicle breakdown, IVM: in vitro maturation. Other abbreviations are as listed in Table 1. a–g: There are significant differences between items with different letters in the same column (P < 0.05). Each treatment was replicated 3–4 times, and each replicate included approximately 30 COCs. (DOCX) [file pone.0312241.s007.docx]

**S7** **Table. Changes in the chromatin configuration during IVM of rat oocytes with the cSN-1 configuration.** GVBD: germinal vesicle breakdown, IVM: in vitro maturation. Other abbreviations are as listed in Table 1. ^a–g^: There are significant differences between items with different letters in the same column (P < 0.05). Each treatment was replicated 3–4 times, and each replicate included approximately 30 COCs.

| Culture time (h) | Number of oocytes | Proportion of oocytes with each chromatin configuration (%) | | |
| --- | --- | --- | --- | --- |
|  |  | cSN-1 | SN-2 | GVBD |
| 0.5 | 63 | 58.97 ± 1.54^a^ | 39.92 ± 1.03^a^ | 1.11 ± 1.11^a^ |
| 1 | 61 | 40.89 ± 1.01^b^ | 52.73 ± 1.61^b^ | 6.38 ± 0.98^b^ |
| 1.5 | 68 | 21.98 ± 0.55^c^ | 35.35 ± 0.36^c^ | 42.67 ± 0.18^c^ |
| 2 | 70 | 8.22 ± 0.97^d^ | 30.67 ± 2.03^d^ | 61.11 ± 1.11^d^ |
| 2.5 | 62 | 0.00 ± 0.00^e^ | 28.87 ± 1.02^d^ | 71.13 ± 1.02^e^ |
| 3 | 74 | 0.00 ± 0.00^e^ | 21.69 ± 0.26^e^ | 78.31 ± 0.26^f^ |
| 3.5 | 82 | 0.00 ± 0.00^e^ | 7.64 ± 1.19^f^ | 92.36 ± 1.19^g^ |
