# Supplementary material for: Relationship between chromatin configuration and maturation ability of rat oocytes in vitro and in vivo
Source: PLoS One. 2025 Feb 13;20(2):e0312241. doi: 10.1371/journal.pone.0312241 (PMC11825056; doi:10.1371/journal.pone.0312241)
Supplement: S8 Table — GVBD: germinal vesicle breakdown, IVM: in vitro maturation. Other abbreviations are as listed in Table 1. a–d: There are significant differences between items with different letters in the same column (P < 0.05). Each treatment was replicated 3–4 times, and each replicate included approximately 30 COCs. (DOCX) [file pone.0312241.s008.docx]

**S8** **Table. Changes in the chromatin configuration during IVM of rat oocytes with the SN-2 configuration.** GVBD: germinal vesicle breakdown, IVM: in vitro maturation. Other abbreviations are as listed in Table 1. ^a–d^: There are significant differences between items with different letters in the same column (P < 0.05). Each treatment was replicated 3–4 times, and each replicate included approximately 30 COCs.

| Culture time (h) | Number of oocytes | Proportion of oocytes with each chromatin configuration (%) | |
| --- | --- | --- | --- |
|  |  | SN-2 | GVBD |
| 0.5 | 76 | 86.82 ± 1.76^a^ | 13.18 ± 1.76^a^ |
| 1 | 47 | 72.77 ± 1.27^b^ | 27.23 ± 1.27^b^ |
| 1.5 | 51 | 41.07 ± 1.79^c^ | 58.93 ± 1.79^c^ |
| 2 | 78 | 8.07 ± 1.19^d^ | 91.93 ± 1.20^d^ |
