# Supplementary material for: Relationship between chromatin configuration and maturation ability of rat oocytes in vitro and in vivo
Source: PLoS One. 2025 Feb 13;20(2):e0312241. doi: 10.1371/journal.pone.0312241 (PMC11825056; doi:10.1371/journal.pone.0312241)
Supplement: S9 Table — GV: germinal vesicle. All other abbreviations are as listed in Table 1. a–b: There are significant differences between items with different letters in the same column (P < 0.05). Each treatment was replicated 3–4 times, and each replicate included approximately 30 COCs. (DOCX) [file pone.0312241.s009.docx]

**S9 Table. Configuration of GV chromatin in healthy and atretic follicles from rats.** GV: germinal vesicle. All other abbreviations are as listed in Table 1. ^a–b^: There are significant differences between items with different letters in the same column (P < 0.05). Each treatment was replicated 3–4 times, and each replicate included approximately 30 COCs.

| Follicle | Number of oocytes | Proportion of oocytes with each chromatin configuration (%) | | | | | | | | |
| --- | --- | --- | --- | --- | --- | --- | --- | --- | --- | --- |
|  |  | NSN (Total) | | | | SN (Total) | | | | |
|  |  | Total | NSN | cNSN | pNSN | Total | pSN-1 | SN-1 | cSN-1 | SN-2 |
| Healthy | 123 | 3.61 ± 0.90^a^ | 0.56 ± 0.56^a^ | 0.65 ± 0.65^a^ | 2.41 ± 0.46^a^ | 96.39 ± 0.90^a^ | 4.80 ± 1.44^a^ | 15.91 ± 0.39^a^ | 28.75 ± 1.65^a^ | 46.92 ± 3.28^a^ |
| Atretic | 100 | 13.14 ± 1.51^b^ | 4.57 ± 1.58^a^ | 4.43 ± 1.29^a^ | 4.14 ± 0.51^a^ | 86.86 ± 1.51^b^ | 3.29 ± 0.38^a^ | 9.43 ± 0.87^b^ | 47.13 ± 3.63^b^ | 27.01 ± 2.89^b^ |
