# Supplementary material for: Relationship between chromatin configuration and maturation ability of rat oocytes in vitro and in vivo
Source: PLoS One. 2025 Feb 13;20(2):e0312241. doi: 10.1371/journal.pone.0312241 (PMC11825056; doi:10.1371/journal.pone.0312241)
Supplement: S10 Table — All abbreviations are as listed in Table 1. (DOCX) [file pone.0312241.s010.docx]

**S10 Table. Chromatin configuration and transcriptional activity of rat oocytes.** All abbreviations are as listed in Table 1.

| Configuration | Oocytes with transcriptional activity | Total | Transcription ratio |
| --- | --- | --- | --- |
| NSN | 86 | 86 | 100.00 ± 0.00 |
| cNSN | 42 | 42 | 100.00 ± 0.00 |
| pNSN | 50 | 52 | 96.88 ± 3.13 |
| pSN-1 | 44 | 58 | 72.16 ± 17.90 |
| SN-1 | 11 | 34 | 27.18 ± 9.62 |
| cSN-1 | 6 | 107 | 4.85 ± 3.05 |
| SN-2 | 0 | 63 | 0.00 ± 0.00 |
